# Barriers Experienced by Community-Dwelling Older Adults Navigating Formal Care: Evidence From an Australian Population-Based National Survey

**DOI:** 10.1177/08982643241263132

**Published:** 2024-06-24

**Authors:** Yuchen Xie, Craig Sinclair, Myra Hamilton, Carmelle Peisah, Jeromey Temple, Kaarin J. Anstey

**Affiliations:** 1 Ageing Futures Institute, 567274 University of New South Wales, Sydney, NSW, Australia; 2 School of Psychology, 441985 University of New South Wales, Sydney, NSW, Australia; 3 6803 Neuroscience Research Australia, Sydney, NSW, Australia; 4 University of Sydney, Sydney, NSW, Australia; 5 Discipline of Psychiatry, 7800 University of New South Wales, Sydney, NSW, Australia; 6 Melbourne School of Population and Global Health, 50066 University of Melbourne, Melbourne, VIC, Australia

**Keywords:** home care, social factors, health services, social support, policy, unmet needs

## Abstract

**Objectives:**

This study aims to identify the relationship between psychosocial factors and unmet needs among community-dwelling older adults who have received or who expect to receive formal home-based aged care services.

**Methods:**

A subsample of the national Survey of Disability, Ageing and Carers was used to examine the prevalence of having any unmet needs among older adults navigating care. We also examined associations between older adults’ psychosocial factors and their unmet needs using logistic regression.

**Results:**

Regression analyses highlighted that perceived social isolation (OR = 1.62, 95% CI: 1.30–2.01), high/very high psychological distress (OR = 2.11, 95% CI: 1.52–2.93), and occasional assistance from informal support (OR = 1.92, 95% CI: 1.22–3.05) were associated with increased odds of having unmet needs, after adjusting for other covariates.

**Discussion:**

Our study suggests that older adults facing psychosocial difficulties or lacking informal support are more likely to encounter barriers in accessing formal care. Future policy should address the psychosocial needs and support networks of older adults.

## Introduction

In the Organisation for Economic Cooperation and Development (OECD) member countries, 68% of individuals aged 65 years and over (i.e., older adults) who are recipients of aged care^1^ receive home-based care (The Organisation for Economic Cooperation and Development, 2021). Most older adults prefer to receive care and support at home rather than in residential care settings (Kasper et al., 2019). With the rapid aging of the population globally, the demand for formal home-based aged care services is increasing. Therefore, ensuring a clear understanding of the pattern of care needs among community-dwelling older adults is imperative.

Home-based aged care services provide assistance with Activities of Daily Living (ADLs) and Instrumental Activities for Daily Living (IADLs) according to older adults’ care needs, to enable them to live independently in their homes as long as possible (Kalánková et al., 2021). There are widely acknowledged barriers to accessing this assistance. One way of measuring such barriers faced by older adults in accessing aged care services is through their self-reported unmet care needs (Hill, 2022; Kalánková et al., 2021). Unmet needs in aged care settings refer to the difference between the care needed and the actual services received by older adults (Kalánková et al., 2021). There are two types of measures for unmet needs. Absolute measures identify individuals who need but receive no support, while relative measures identify those who need and receive support but judge existing support to be insufficient (Spiers et al., 2022). Unmet care needs can lead to a range of negative outcomes among older adults, including adverse consequences such as medication errors (Allen et al., 2014; Berridge & Mor, 2018), falls (Marrero et al., 2019), mortality (He et al., 2015), and admission to residential care (Chen & Thompson, 2010). Previous research has found that several individual characteristics such as gender, ethnicity, type of primary carer, and living alone are associated with expressed unmet needs among older adults (Liu et al., 2012; Wilkinson-Meyers et al., 2014; Zhu, 2015).

A limited number of studies have examined associations between psychosocial factors and barriers to accessing care (Quail et al., 2011; Shaw et al., 2017), indicating the importance of research into psychosocial factors associated with unmet needs. Furthermore, a recent review found that social support contributed to aged care navigation (Xie et al., 2024). However, little is known about the relationship between social connectedness and unmet needs among older adults (Chamberlain et al., 2023). Research is needed to understand how these psychosocial factors such as access to informal supports or the related, but distinctly subjective concept of social isolation shape care access and unmet needs (Gilmour, 2018; Quail et al., 2011; Zhu, 2015). Understanding the relationship between social connectedness and unmet needs is particularly important due to rising levels among older adults of (i) social isolation and loneliness (Gardiner et al., 2018) exacerbated by the COVID pandemic (Hwang et al., 2020); (ii) living alone (Nyqvist et al., 2017); (iii) childlessness and divorce (Hamilton et al., 2020); and (iv) geographical separation from family with increasing mobility and migration (King et al., 2014). In addition, older people from Culturally and Linguistically Diverse (CALD) Backgrounds are among the most vulnerable groups at risk of social isolation (Rao et al., 2006; Wright-St Clair et al., 2017). Evidence also suggests that social isolation is influenced by cultural background, language barriers, and the social connection and support networks of older adults (Park et al., 2019; Rao et al., 2006; Wu & Penning, 2015). A recent report in Australia suggested that people from CALD backgrounds who were socially isolated had increased risk of utilizing healthcare services (Barr et al., 2020). This indicates a need to consider the cultural and language factors, and the social isolation among older adults within the context of accessing aged care services. This study intends to address these research gaps, by investigating the relationship between these psychosocial factors and barriers to navigating formal aged care services among community-dwelling older Australians by measuring their reported unmet care needs.

Using data from the 2018 Australian Survey of Disability, Ageing and Carers (SDAC), this study aims to examine the prevalence and psychosocial-related correlates of unmet needs for formal home-based aged care services among community-dwelling older adults (Australian Bureau of Statistics, 2018; Steering Committee for the Review of Government Service Provision, 2019). In this study, we focus on relative unmet needs as the measure of the barriers to navigating and accessing formal home-based aged care services by community-dwelling older adults.

Our primary objective is to examine the association between community-dwelling older adults’ psychosocial factors (e.g., perceived social isolation, English language proficiency, and access to informal assistance) and having any unmet needs for formal home-based aged care services. Our secondary objective is to estimate the prevalence of unmet needs among community-dwelling older Australians who have received and those who expect to receive formal home-based aged care services.

## Methods

### Conceptual Framework

Andersen’s behavioral model was used as a conceptual framework to inform the selection of variables in our analysis (Andersen, 1995). The original Andersen model categorizes individual characteristics as predisposing, need-based, and enabling factors. Predisposing factors are demographic characteristics such as age, gender, and socio-economic characteristics; need-based factors refer to an individual’s perceived or evaluated functional capacities such as general health status or disability status; enabling factors include individual-level (e.g., knowledge and family support) and community resources (e.g., personnel and facilities) (Andersen, 1995). In Andersen’s expanded model used in aged care settings, psychosocial factors were added as predisposing factors (Bradley et al., 2002).

### Data

Data used for this study are from the Survey of Disability, Ageing and Carers (SDAC) conducted by the Australian Bureau of Statistics (ABS) from July 2018 to March 2019. SDAC is an Australian population-based survey using a repeated cross-sectional design to collect information on people with disability, older people (i.e., defined as those aged 65 years and older in SDAC), and informal carers who assist older people or people with disability.

The SDAC survey collected data from people living both in households and institutional settings (Australian Bureau of Statistics, 2018). The households include houses, flats, home units, and self-care components of retirement villages (i.e., community-dwelling). The households are selected randomly from a stratified, multi-stage area sampling technique developed by the ABS (Australian Bureau of Statistics, 2018). Basic demographic and socio-economic information for all household members was provided by a responsible adult (i.e., someone likely to know the most about fellow occupants and can answer questions without reference to other household members) or personal interviews if preferred from each selected household. The responsible adults were asked screening questions to determine the need for additional personal interviews relevant for primary carer(s) and/or people with disability/aged over 65 years and above in the same household.

In this study, home-based aged care refers to formal home-based aged care services received by community-dwelling older adults. The formal care services collected by the SDAC survey include assistance from both government and private care providers.

### Analytical Sample

Data for this study were obtained through an agreement between the ABS and Universities Australia as a basic microdata Confidentialised Unit Record File (CURF). Ethics approval for this study was obtained from the University of New South Wales. Person-level and household-level datasets were used in this study and analyzed using Stata version 17.0. The person-level dataset consists of 65,487 individuals. The household-level dataset includes information on 21,949 households.

**Figure 1.**
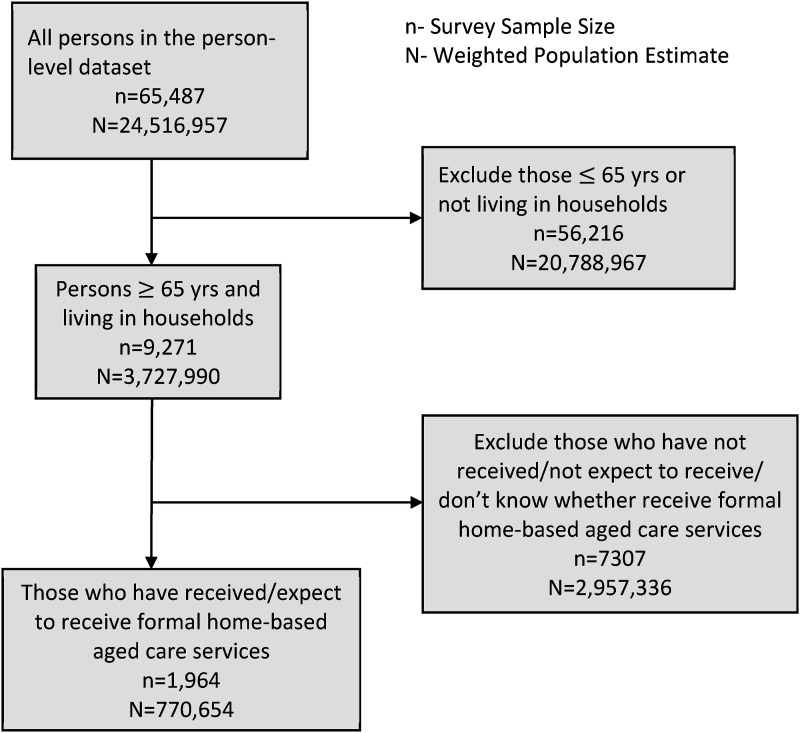
Overview of the analytical sample from the 2018 Survey of Disability, Ageing and Carers. Source: Basic microdata Confidentialised Unit Record File from the Australian Bureau of Statistics.

The person-level dataset was the main dataset used to address our research objectives. We aimed to access data from community-dwelling older adults who have received or who expect to receive formal home-based aged care services from at least one of ten broad areas of activity. To reflect this population of interest, we first selected a subset of people aged 65 years and above living in households from the person-level dataset in SDAC (*n* = 9271 in Figure 1). We further selected those who have received or who expect to receive formal assistance from at least one of the ten broad areas of activity for at least six months (*n* = 1964). These are the community-dwelling older adults with reported needs for formal home-based aged care services.

The person-level and household-level datasets were merged (*n* = 1964) by the authors through household identifiers to obtain the weekly household income information used for our data analyses. Details of the selection of our analytical sample are illustrated in Figure 1.

### Outcomes: Any Unmet Needs for Formal Home-Based Aged Care Services

In this study, unmet needs for formal home-based aged care services are identified for an individual when they either do not receive the necessary assistance, or receive less than what is required, from one or more of the ten broad areas of activity, including mobility, self-care, communication, healthcare, cognitive or emotional tasks, household chores, property maintenance, meal preparation, reading or writing, and transport (Australian Bureau of Statistics, 2018). As mentioned earlier, the unmet needs in this study refer to the relative unmet needs. In our analytical sample (*n* = 1964), 643 had any unmet needs for formal assistance (i.e., defined as cases in this study). The remaining 1321 did not report having any unmet needs for formal assistance and were defined as controls.

Respondents who indicated having unmet needs for at least one of the ten broad areas of activity (*n* = 643) were also asked to provide all reasons for not receiving (any or more) help with any of the areas of activity. The respondents could select one or more categories to indicate the reasons for having any unmet needs from the following: Did not know of service; need not important enough; won’t ask/pride; unable to arrange service; no services available; not eligible for service; service costs too much; service doesn’t provide sufficient hours; and other reason.

The above response categories from the respondents indicate specific barriers experienced by older adults in accessing care. Therefore, having any unmet needs (i.e., reporting unmet needs for at least one broad areas of activity) was used as an indicator for experiencing barriers in accessing formal home-based aged care services.

### Covariates: Measurement of Individual Characteristics

Based on Andersen’s model, we included other individual characteristics from SDAC that might be associated with unmet needs. These measures included English language proficiency, perceived social isolation, access to informal assistance, and psychological distress, all described in greater detail below.

#### Social Isolation

The SDAC questionnaire does not include a direct measurement of social isolation, but there is one question that captures a subjective measure of social isolation (i.e., perceived social isolation). Respondents were asked: “Would you like to have more contact with family or friends (who do not live with you)?” The answer to this question separates respondents into three groups: (1) those who want more contact with family or friends, (2) those who do not want more contact with family or friends, and (3) those who have no family or friends. We combined groups (1) and (3) into one category of those who experienced perceived social isolation, while group (2) were those who did not report perceived social isolation.

#### English Language Proficiency

The ABS has developed several cultural and language indicators to measure the cultural diversity of the population. There are several measures used by ABS to indicate a person’s preferred language spoken at home, such as “main language spoken at home.”

In the ABS survey, all the respondents were categorized into two groups: (1) those whose main language spoken at home is English, and (2) those whose main language spoken at home is not English. Those whose main language is not English were further categorized in their proficiency in spoken English: very well, well, not well, and not at all.

In this study, a new variable called “English language proficiency” was derived from “proficiency in spoken English” and “main language spoken at home” into five categories: language spoken is English (reference group) and other language spoken at home with four levels of proficiency in spoken English as very well, well, not well, and not at all.

#### Psychological Distress

The Kessler Psychological Distress 10-item Scale (i.e., K10) has been widely used as a simple measure of psychological distress through respondents’ self-report of their emotional state in the past four weeks. The K10 was included in the SDAC questionnaire. We used the K10 categorized score and recategorized the variable into four categories: (1) low; (2) moderate; (3) high/very high; and (4) not asked/unable to determine.

#### Access to Informal Assistance

To measure the access to informal assistance for older adults, we used the variable “Frequency of informal assistance received with at least one activity.” This measure comprised ten groups of responses: one response item indicated “Do not receive any informal assistance,” while the other nine indicated they received some form of informal assistance with various frequencies (e.g., six or more times a day; three to five times a day; … and less than once a year). We recategorized these nine responses as “occasional assistance,” “weekly/monthly assistance,” “regular daily assistance,” and “high daily assistance.”

### Analytical Strategy

We assessed the prevalence of predisposing (i.e., age, sex, highest education, perceived social isolation, and psychological distress), enabling (i.e., household income, English language proficiency, region of residence, and access to informal assistance), and needs-related (i.e., disability status) factors among (i) all community-dwelling older adults ≥65 years (*n* = 9271) and (ii) those who have received or who expect to receive formal home-based aged care services for at least six months (*n* = 1964). Furthermore, we examined the proportions of different reasons for having unmet needs among those with reported needs for formal home-based aged care services (*n* = 643).

Our inferential analytic sample to detect correlates of unmet needs was restricted to those who have received or who expect to receive formal home-based aged care services for at least six months (*n* = 1964). Specifically, the associations between the psychosocial factors of older adults and their unmet needs were assessed using univariable and multivariable logistic regression models. In the logistic regression models, the outcome variable—unmet needs for formal home-based aged care services—is categorized into having any unmet needs (i.e., the cases) and having no unmet need (i.e., the controls).

The ABS provides a population inverse probability weight alongside 60 replicate weights to calculate corrected standard errors and 95% confidence intervals (CIs) for the prevalence estimates and regression coefficients. We utilized the Jackknife repeated replication method to account for complex sample design features utilizing the population and replicate weights (Bell, 2000). The Archer-Lemeshow test (Archer & Lemeshow, 2006) was conducted to assess the goodness-of-fit of the regression model. Model assumptions were assessed using the variance inflation factor (VIF), and condition numbers (Mason & Perreault, 1991).

## Results

### Descriptive Statistics

In 2018/2019, it was estimated that over 3.7 million older Australians aged 65 or older living in households (i.e., community-dwelling older adults) based on the sample of 9271 older adults in the SDAC study. Of these older adults, 1964 of them (20.7%; weighted population estimate: *N* = 770,654) have received or expect to receive formal home-based aged care services (i.e., those with formal assistance needs with at least one of the ten broad areas of activity for at least six months). Furthermore, among the 1964 adults with formal assistance needs, 643 of them (32.4%; weighted population estimate: *N* = 249,715) reported having any unmet needs.

Table 1 presents the demographic characteristics of the community-dwelling older adults (*n* = 9271) and the subset of those who have received or who expect to receive formal home-based aged care services (*n* = 1964). Most community-dwelling older adults (59%) were in the age group 65–74 years. Of those who have received or who expect to receive formal home-based aged care services (hereafter mentioned as “care”), more than 46% were aged 80 years and over. Among all community-dwelling older adults, there were slightly more females (52.5%) than males. Almost 26% of them reported obtaining a bachelor’s degree or higher. Of those who have received or who expect to receive care, almost 64% of them were female. Most community-dwelling respondents (who may or may not receive care) reported speaking English (almost 90%) at home.

**Table 1.** Demographic Characteristics of Community-Dwelling Older Australians (Aged 65 or Older), 2018/2019, Weighted Percentages.

	Percentages of Older Adults (95% CI)	
	Community-Dwelling Older Adults ≥65 Years (*n* = 9271, *N* = 3,727,990)	Community-Dwelling Older Adults ≥65 Years Who Have Received or Who Expect to Receive Formal Home-Based Aged Care Services (*n* = 1964, *N* = 770,654)
*Predisposing/psychosocial factors*		
Age		
65–69 years	31.98 (31.95–32.02)	15.73 (14.22–17.36)
70–74 years	27.14 (27.11–27.16)	19.13 (17.46–20.93)
75–79 years	18.42 (18.41–18.44)	19.03 (17.46–20.70)
80–84 years	12.18 (12.16–12.19)	19.97 (18.36–21.69)
85 years and over	10.28 (10.26–10.30)	26.14 (24.45–27.91)
Sex		
Male	47.50 (47.49–47.52)	36.14 (33.97–38.37)
Female	52.50 (52.48–52.51)	63.86 (61.63–66.03)
Highest education		
Year 12 or less	51.46 (50.37–52.56)	59.76 (57.09–62.37)
Certificate	18.20 (17.33–19.11)	15.58 (13.55–17.84)
Bachelor’s degree or higher	25.48 (24.49–26.48)	20.53 (18.51–22.70)
Not determined	4.86 (4.48–5.27)	4.14 (3.26–5.24)
Perceived social isolation		
Wants more contact with family/friends	26.27 (25.00–27.57)	32.16 (29.66–34.76)
Does not want more contact with family/friends	73.54 (72.23–74.81)	67.66 (65.05–70.17)
Has no family/friends	0.19 (0.09–0.39)	0.18 (0.06–0.50)
Psychological distress		
Low	65.37 (64.11–66.61)	41.71 (39.56–43.90)
Moderate	15.70 (14.74–16.71)	24.73 (22.36–27.26)
High/very high	9.40 (8.62–10.25)	21.13 (18.96–23.48)
Unable to determine/not asked	9.53 (8.78–10.33)	12.43 (10.78–14.28)
*Enabling factors*		
Household income (weekly)		
1st to 3rd decile (≤383 AUD)	39.53 (38.16–40.92)	47.70 (44.67–50.75)
4^th^ to 6^th^ decile (384–950 AUD)	24.32 (22.87–25.83)	20.29 (17.81–23.03)
7th to 10th decile (≥951 AUD)	10.73 (9.77–11.77)	5.62 (4.29–7.34)
Not known	25.42 (24.03–26.87)	26.38 (23.90–29.03)
English language proficiency		
Main language spoken at home is English	89.65 (88.70–90.52)	89.83 (87.77–91.57)
Main language spoken at home is not English		
Very well	2.11 (1.71–2.60)	1.58 (1.00–2.48)
Well	3.76 (3.25–4.36)	3.07 (2.14–4.39)
Not well	3.41 (2.91–3.99)	4.56 (3.48–5.96)
Not at all	1.07 (0.76–1.50)	0.96 (0.53–1.74)
Region of residence		
Major cities of Australia	66.95 (65.91–67.98)	67.63 (64.99–70.16)
Inner regional Australia	22.67 (21.22–24.20)	23.59 (20.94–26.46)
Other areas	10.37 (9.22–11.65)	8.78 (7.27–10.57)
Access to informal assistance		
Do not receive any informal assistance	27.57 (25.87–29.34)	32.51 (30.06–35.06)
Occasional assistance	5.31 (4.45–6.31)	4.60 (3.64–5.79)
Weekly/monthly assistance	36.64 (35.11–38.19)	31.53 (29.40–33.74)
Regular daily assistance	14.03 (12.56–15.65)	13.97 (12.07–16.12)
High daily assistance	16.45 (14.92–18.12)	17.39 (15.41–19.57)
*Need-related factors*		
Disability status		
Without disability	52.65 (51.33–53.95)	11.36 (9.89–13.00)
Disability but no limitation in core activities	4.91 (4.45–5.42)	2.26 (1.70–3.01)
Disability with mild limitation	20.84 (19.79–21.92)	24.56 (22.52–26.72)
Disability with moderate limitation	7.78 (7.08–8.54)	18.10 (16.47–19.85)
Disability with severe limitation	6.03 (5.47–6.66)	17.89 (15.91–20.06)
Disability with profound limitation	7.80 (7.23–8.40)	25.83 (23.71–28.07)

*Notes:* all percentages are weighted to account for complex survey design features.

This table presents the characteristics of study sample by predisposing, enabling, and needs-related factors, Community-dwelling older Australians (aged 65 or older), Australia, from the Survey of Disability, Ageing and Carers.

Around 53% of community-dwelling older adults did not have disability, while among those receiving or expecting to receive care, more than 88% had disability. Higher psychological distress levels (i.e., high/very high distress level) were observed among older adults receiving or expecting to receive care (21.13%) than the broader community-dwelling sample (9.40%). Similar trends were observed in perceived social isolation. More older adults who have received or who expect to receive care reported wanting to have more contact with family or friends than the wider sample of community-dwelling older adults (32.16% vs. 26.27%).

In addition, Table 1 illustrates the distribution of informal assistance levels, ranging from no assistance to high levels of daily support. Specifically, 27.57% of community-dwelling older adults reported not receiving any informal assistance, while 36.64% received weekly/monthly informal assistance. Compared to the community-dwelling older adults, a higher proportion of those who have received or who expect to receive care reported not having any informal assistance (32.51% vs. 27.57%).

### Reasons for Having Any Unmet Needs

**Figure 2.**
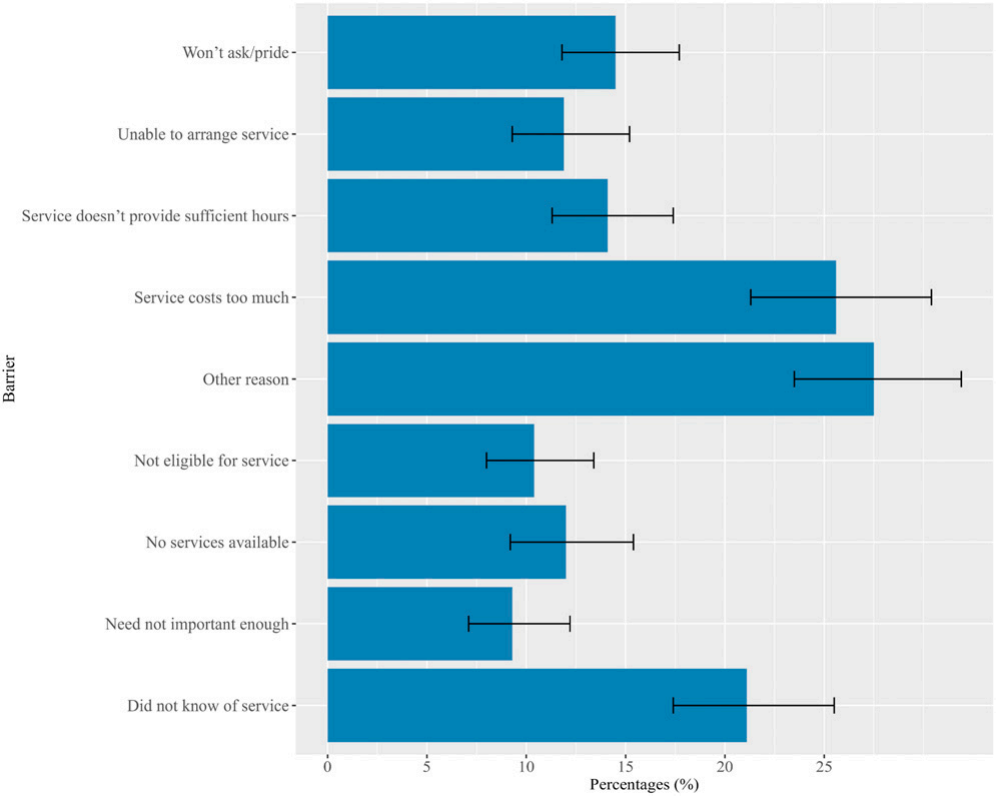
Reasons for having any unmet needs among community-dwelling older adults (aged 65 or older) for formal home-based aged care services (*n* = 643, *N* = 249,715). (Note: The error bars represent the 95% confidence interval for each of the estimated proportion for a reason of having any unmet needs).

Among older adults who reported having any unmet needs for formal home-based aged care services (*n* = 643), the top three known reasons were “service costs too much” (25.6%), “did not know of service” (21.1%), and “won’t ask/pride” (14.5%). The most frequently reported reasons were “other reason” (27.5%). The “other reason” was not unpacked in the SDAC survey. Other reported reasons included “service does not provide sufficient hours” (14.1%), “no service available” (12.0%), “unable to arrange service” (11.9%), etc. All the reasons for having any unmet needs from formal home-based aged care services are described in Figure 2.

### Multivariable Regression Results

Table 2 shows the odds ratios of any unmet needs for formal home-based aged care services among older Australians with respect to different types of factors described in the extended Andersen framework. Model 1 included demographic and psychosocial variables (i.e., age, sex, highest education, perceived social isolation, and psychological distress). In Model 2, we added variables related to individual and family resources (i.e., household income, English language proficiency, region of residence, and access to informal assistance). Model 3 further added variables on need-related factors (i.e., disability status).

**Table 2.** Adjusted Odds Ratios Relating to Selected Predisposing, Enabling, and Needs-Related Characteristics to Any Unmet Needs Among Community-Dwelling Older Adults (Aged 65 or Older) Who Have Received or Who Expect to Receive Formal Home-Based Aged Care Services (*n* = 1964, *N* = 770,654).

	Model 1		Model 2		Model 3	
	Odds Ratio	95% CI	Odds Ratio	95% CI	Odds Ratio	95% CI
*Predisposing/psychosocial characteristics*						
Age						
65–69 years	Ref		Ref		Ref	
70–74 years	1.00	0.68–1.45	0.99	0.67–1.47	1.00	0.67–1.48
75–79 years	1.39	0.91–2.10	1.38	0.89–2.13	1.35	0.87–2.10
80–84 years	1.01	0.68–1.50	0.95	0.63–1.42	0.94	0.62–1.43
85 years and over	0.88	0.63–1.24	0.84	0.59–1.20	0.80	0.55–1.16
Sex						
Male	Ref		Ref		Ref	
Female	1.63***	1.27–2.10	1.62***	1.26–2.08	1.67***	1.28–2.16
Highest education						
Year 12 or less	Ref		Ref		Ref	
Certificate	1.52*	1.06–2.16	1.54*	1.07–2.22	1.53*	1.06–2.21
Bachelor’s degree or higher	1.51**	1.14–2.01	1.57**	1.15–2.15	1.62**	1.18–2.23
Not determined	1.05	0.64–1.74	1.08	0.65–1.80	1.11	0.67–1.82
Perceived social isolation						
No	Ref		Ref		Ref	
Yes	1.68***	1.36–2.07	1.66***	1.34–2.06	1.62***	1.30–2.01
Psychological distress						
Low	Ref		Ref		Ref	
moderate	1.83***	1.33–2.53	1.83***	1.32–2.53	1.64**	1.15–2.32
High/very high	2.61***	1.93–3.53	2.51***	1.85–3.41	2.11***	1.52–2.93
Unable to determine/not asked	2.49***	1.71–3.63	2.29**	1.43–3.65	2.05**	1.28–3.29
*Enabling factors*						
Household income (weekly)						
1st to 3rd decile (≤383 AUD)			Ref		Ref	
4^th^ to 6^th^ decile (384–950 AUD)			0.73*	0.55–0.98	0.76	0.57–1.01
7th to 10th decile (≥951 AUD)			0.86	0.58–1.26	0.91	0.61–1.35
Not known			0.92	0.68–1.24	0.94	0.69–1.28
English language proficiency						
Main language spoken at home is English			Ref		Ref	
NE: Very well			0.73	0.28–1.88	0.80	0.31–2.09
NE: Well			1.10	0.49–2.47	1.04	0.47–2.32
NE: Not well			0.87	0.50–1.54	0.85	0.47–1.52
NE: Not at all			1.40	0.54–3.59	1.36	0.53–3.50
Region of residence						
Major cities of Australia			Ref		Ref	
Inner regional Australia			1.1	0.80–1.52	1.11	0.80–1.53
Other areas			1.14	0.81–1.61	1.16	0.81–1.65
Access to informal assistance						
Do not receive any informal assistance			Ref			
Occasional assistance			2.05**	1.31–3.20	1.92**	1.22–3.05
Weekly/monthly assistance			1.23	0.92–1.65	1.05	0.76–1.45
Regular daily assistance			1.24	0.85–1.81	0.98	0.64–1.50
High daily assistance			1.48	0.97–2.25	1.11	0.68–1.81
*Need-related factors*						
Disability status						
Without disability					Ref	
Disability but no limitation in core activities					0.60	0.20–1.77
Disability with mild limitation					1.52	0.94–2.46
Disability with moderate limitation					2.36**	1.25–4.48
Disability with severe limitation					2.61**	1.50–4.54
Disability with profound limitation					2.36**	1.33–4.18

*Notes:* *p < .05, **p < .01, ***p < .001.

Ref: reference group.

NE: main language spoken at home is not English.

All percentages are weighted to account for complex survey design features.

Model 1 was adjusted demographic and psychosocial factors, including age, sex, highest education, perceived social isolation, and psychological distress; Model 2 was further adjusted for enabling factors, including household income, English language proficiency, region of residence, and access to informal assistance, whereas Model 3 was further adjusted for need-related factors (i.e., disability status).

Being female was associated with 62–67% increased odds of having any unmet needs than males in all three models. While the oldest old age group (85 years and over) was negatively associated with having any unmet needs in the univariable model (OR = 0.63, 95% CI: 0.45–0.88), this association was no longer statistically significant across the multivariable models. Higher education was a significant factor positively associated with the odds of having any unmet needs across all three models. The odds of having any unmet needs increased by 51–62% for older adults who held a bachelor’s or higher degree than those who completed Year 12 or less.

Psychosocial factors were significantly associated with having any unmet needs in all three models. The odds of having any unmet needs are 2.11 times higher for older adults with high or very high psychological distress than those with low distress (in Model 3). Similarly, another influential psychosocial factor was perceived social isolation, which led to a significant increase in the odds of having any unmet needs relative to those who do not report perceived social isolation (62%–68% across all three models).

Additional analysis of enabling factors indicated that household income and access to informal assistance play a significant role in having any unmet needs among older adults (Model 2). The odds of having any unmet needs were 27% lower among individuals in the 4^th^ to 6^th^ decile of household income (OR = 0.73, 95% CI: 0.55–0.98) compared to those in the 1^st^ to 3^rd^ decile. However, no statistically significant difference was observed in the odds of having any unmet needs between individuals in the 7^th^ to 10^th^ decile of household income and the reference group (OR = 0.86, 95% CI: 0.58–1.26).

Regarding access to informal assistance, older adults who reported receiving informal assistance occasionally (OR = 2.05, 95% CI: 1.31–3.20) had significantly higher odds of having unmet needs relative to those who did not receive any informal assistance. However, no significant differences were observed in the odds of having any unmet needs between those receiving informal assistance in other frequencies, including weekly/monthly assistance, and regular daily assistance (1–2 times/day) to the same reference group. The association between access to informal assistance and having any unmet needs remained significant in Model 3.

In Model 3, while adjusting all other variables in the previous two models, we added the disability status as a need-related factor. Similar findings to our univariable model results, we found that older adults with disability demonstrated significantly higher odds of having any unmet needs with increasing levels of limitation in core activities compared to those without disability, as indicated by the higher odds ratios observed for moderate (OR = 2.36, 95% CI: 1.25–4.48), severe (OR = 2.61, 95% CI: 1.50–4.54), and profound limitations (OR = 2.36; 95% CI: 1.33–4.18). However, older adults with disability but no limitation (OR = 0.60, 95% CI: 0.20–1.77) or those with disability but mild limitation in core activities (OR = 1.52, 95% CI: 0.94–2.46) did not show a significant difference in the odds of having any unmet needs relative to the same reference group.

The result of the Archer-Lemeshow test suggested that the model fit was acceptable [*p*-value = .147]. Further post estimation analysis of variance inflation factors (mean VIF = 1.71) and condition number (condition number = 15.02) diagnostics confirmed the model was robust to multicollinearity.

A summary of univariable logistic regression models for predisposing, enabling, and needs-related factors is presented in the Supplementary Table.

## Discussion

To the best of our knowledge, this is the first study in Australia to investigate the extent of any unmet needs in receiving formal home-based aged care services among community-dwelling older adults using data from a population-based national survey. In this study, any unmet needs as outcome measures refer to formal assistance from the ten broad areas of activity, which can be classified into either ADLs or IADLs assistance (Edemekong et al., 2019). Although we did not analyze the two types of assistance separately, our study adds to the existing literature on unmet needs for formal assistance with ADLs and IADLs among community-dwelling older adults.

### Reasons for Having Unmet Needs

A key contribution of this study is that we examined the diverse reasons (i.e., barriers) reported by community-dwelling older adults with any unmet needs when seeking formal home-based aged care services. One-quarter (25.6%) of older adults with any unmet needs identified expensive services as a significant barrier, whereas a fifth (21.1%) reported a lack of awareness regarding available services. These findings highlight the importance of implementing strategies to increase knowledge and awareness among older adults about such services. Interestingly, around 15% of older adults cited “won’t ask/pride” as the reason for not receiving formal assistance. The reluctance to seek help when accessing formal care may imply the presence of social stigma or self-ageism toward help-seeking behavior (de São José & Amado, 2017; Teo et al., 2022). It may be that older adults associate asking for help with a perceived loss of independence or an acknowledgment of aging. Efforts should also be made to address the financial burden associated with accessing services. Furthermore, a significant portion of older adults (27.5%) reported “Other reason” as the barrier to not receiving care, underscoring the need for future research to explore these unidentified factors.

### Predisposing Factors

Our findings underscore the importance of psychosocial factors in reported unmet needs for home-based care among older adults. The association between higher levels of psychological distress among older adults and increased unmet care needs is concerning. A significant proportion (45.86%) of older adults receiving formal home-based aged care services reported moderate and above psychological distress, with one-fifth reporting high to very high levels of distress. Similarly, perceived social isolation among older adults was highly associated with having any unmet care needs. These associations remained significant even after adjusting for other predisposing, enabling, and need-related factors in the Andersen model. These results were consistent with a recent study examining the relationship between loneliness and unmet healthcare needs among middle-aged and older adults (Chamberlain et al., 2023). Furthermore, a cross-sectional study conducted among older American Indians living in the community indicated that lower levels of social support were associated with having unmet needs (Schure et al., 2015). Our findings on psychosocial distress echoed a Canadian study by Quail et al. (2011), who found a statistically significant association between IADL unmet needs and psychological distress in community-dwelling older women.

Being female was associated with increased odds of having any unmet care needs, even after adjusting for other covariates. This finding is consistent with a previous study reporting that females in Canada and Austria were more likely to express unmet healthcare needs (Tadiri et al., 2021). Perhaps the most interesting finding was the relationship between the highest education obtained by older adults and their unmet needs. Prior research indicated that education status influenced unmet needs among older adults differently based on their depression status (Meng et al., 2021). Our study showed that higher education was associated with increased odds of having any unmet needs, which accords with a recent study from Canada (Chamberlain et al., 2023). These findings may be due to more educated individuals having higher expectations of care or being able to better articulate their unmet care needs. However, more research is needed to investigate the effects of education on unmet needs.

### Enabling Factors

Our study also examined the extent to which the older adult’s access to informal assistance may influence their unmet needs. Our findings suggested that occasional assistance from informal support might in fact increase the odds of having any unmet needs for formal home-based aged care services. However, we did not observe such an association between higher frequencies of receiving informal assistance and unmet needs. These findings suggest that the informal assistance provided to older adults may not adequately cater to their unique care needs, possibly due to the constraints experienced by informal carers in fulfilling these needs (Brinkmann et al., 2021). A notable concern arises for older adults with only occasional informal assistance, as they may not receive sufficient assistance from either formal services or family/social support networks, potentially leaving them in a more vulnerable position with higher levels of unmet needs. Our findings might also reflect relational satisfaction with family carers, although this needs to be tested. Future research needs to investigate the nuanced role of family support in care provision.

Another finding that stands out from our study was the lack of impact of English language proficiency on older adults’ unmet needs. The language barrier has been identified as a major obstacle to accessing healthcare information and seeking support in primary care settings (Jaeger et al., 2019; Lim et al., 2019). However, our study did not find an association between English language proficiency and having any unmet needs, suggesting that this may not constitute a direct barrier for older adults in accessing formal home-based aged care services. A possible explanation is that those who spoke a language other than English may be less informed about their care options, hence having lower expectations or less capacity to communicate their unmet needs. Another possible interpretation of this finding is that English language proficiency as a sole indicator may not accurately represent the cultural and linguistic challenges faced by older adults with CALD backgrounds. In Australia, the latest statistics show that 27.6% of home-based recipients’ preferred language is not English (Australian Institute of Health and Welfare, 2023). Hence, future studies are needed to examine how other cultural and linguistic indicators might influence the access of older adults with CALD backgrounds to home-based aged care services.

Finally, we did not find a statistical association between any unmet needs and residence in regional areas, which is inconsistent with another study (Ma et al., 2023). Future research is needed to evaluate the effects of rural residency on unmet needs for home-based aged care services among older adults.

#### Limitations

Since we are particularly interested in older adults who navigate formal home-based aged care services, our analysis used relative unmet need measures. Relative unmet needs are driven by expectations of care, which is a judgment on the adequacy of support received (Spiers et al., 2022). Therefore, our figures may not include those in need of formal home-based aged care services but receive no support (i.e., those with absolute unmet needs). Future research examining unmet needs for home-based aged care should include both absolute and relative measures. Furthermore, the survey questionnaire in SDAC does not differentiate between respondents who have received formal home-based aged care and those who expect to receive it. Hence, we are unable to analyze the differences between those two groups. In addition, the SDAC survey does not include a direct measure of social connectedness. Therefore, the perceived social isolation in our study might not appropriately represent the social isolation status of the older adults. Future studies should consider direct measures of social isolation among older adults and examine its effects on aged care services utilization. Finally, the cross-sectional study design of the SDAC does not establish causal relationships when interpreting findings.

#### Conclusion

Our findings suggested that community-dwelling older adults struggling with psychosocial issues (e.g., psychological distress and perceived social isolation), or experiencing limited informal assistance from their support networks might encounter more challenges in accessing formal aged care services. Future aged care reform needs to emphasize the importance of psychosocial needs and the support network of older adults to better address their unmet care needs.

## Supplemental Material

Supplemental Material - Barriers Experienced by Community-Dwelling Older Adults Navigating Formal Care: Evidence From an Australian Population-Based National Survey Supplemental Material for Barriers Experienced by Community-Dwelling Older Adults Navigating Formal Care: Evidence From an Australian Population-Based National Survey by Yuchen Xie, Craig Sinclair, Myra Hamilton, Carmelle Peisah, Jeromey Temple, and Kaarin J. Anstey in Journal of Aging and Health.

## Note

1. Aged care versus long-term care: In this paper, we use the term “aged care” instead of “long-term care.” We have chosen this terminology because “aged care” is specifically focused on meeting the needs of older adults, while “long-term care” focuses more broadly on people with chronic conditions and disabilities, regardless of age.
