# Supplementary material for: Barriers Experienced by Community-Dwelling Older Adults Navigating Formal Care: Evidence From an Australian Population-Based National Survey
Source: J Aging Health. 2024 Jun 24;37(7-8):479–91. doi: 10.1177/08982643241263132 (PMC12179402; doi:10.1177/08982643241263132)
Supplement: Supplemental Material - Barriers Experienced by Community-Dwelling Older Adults Navigating Formal Care: Evidence From an Australian Population-Based National Survey [file sj-pdf-1-jah-10.1177_08982643241263132.pdf]

Supplementary Table: Univariable Logistic Regression Models: Odds Ratios Relating to Selected Predisposing, Enabling, and Needs-related Characteristics to Any Unmet Needs among Community-dwelling Older Adults (Aged 65 or Older) Who Have Received or Expect to Receive Formal Home-based Aged Care Services (n=1964, N=770,654)

	Odds ratio	95% CI
Predisposing/psychosocial characteristics		
**Age**		
65-69 years	Ref	
70-74 years	0.82	0.57-1.19
75-79 years	1.11	0.75-1.64
80-84 years	0.78	0.52-1.16
85 years and over	0.63**	0.45-0.88
**Sex**		
Male	Ref	
Female	1.35**	1.08-1.67
**Highest education**		
Year 12 or less	Ref	
Certificate	1.36	.99-1.86
Bachelor's degree or higher	1.37*	1.04-1.79
Not determined	0.96	0.60-1.53
**Perceived social isolation**		
No	Ref	
Yes	1.95***	1.60- 2.38
**Psychological distress**		
Low	Ref	
Moderate	1.89***	1.37-2.60
High/very high	2.75***	2.03-3.72
Unable to determine/Not Asked	2.27***	1.60-3.22
Enabling factors		
**Household income (weekly)**		
1st to 3rd decile (≤ 383 AUD)	Ref	

4th to 6th decile ($384-$950 AUD)	0.76	0.58-1.00
7th to 10th decile (≥$951 AUD)	1.13	0.74-1.73
Not known	0.94	0.72-1.23
**English language proficiency**		
Main language spoken at home is English	Ref	
NE: Very well	.088	0.34-2.28
NE: Well	1.27	0.60-2.69
NE: Not well	1.03	0.62-1.72
NE: Not at all	1.73	0.67-4.46
**Region of residence**		
Major cities of Australia	Ref	
Inner regional Australia	1.10	0.83-1.45
Other areas	1.13	0.79-1.63
**Access to informal assistance**		
Do not receive any informal assistance	Ref	
Occasional assistance	1.87**	1.25-2.79
Weekly/Monthly assistance	1.22	0.92-1.60
Regular daily assistance	1.38	0.96-1.98
High daily assistance	1.75**	1.27-2.43
Need-related factors		
**Disability status**		
Without disability	Ref	
Disability but no limitation in core activities	0.68	0.25-1.88
Disability with mild limitation	1.61*	1.00-2.58
Disability with moderate limitation	2.94***	1.66-5.21
Disability with severe limitation	3.21***	1.99-5.18
Disability with profound limitation	2.97***	1.93-4.56

Notes: *p<.05, **p<.01, ***p<.001.

Ref: reference group

All percentages are weighted to account for complex survey design features.
